# Factors associated with recurrence in patients with oral cancer in Mongolia

**DOI:** 10.1186/s12885-024-12118-8

**Published:** 2024-03-20

**Authors:** Oyuntsetseg Davaatsend, Munkhdul Altannamar, Mandukhai Ganbat, Urjinlkham Jagdagsuren

**Affiliations:** 1 Department of Maxillofacial Surgery, School of Dentistry, Mongolian National University of Medical Sciences, Ulaanbaatar, Mongolia; 2 Department of Maxillofacial Surgery, School of Dentistry, Ach Medical University, Ulaanbaatar, Mongolia; 3 Department of Epidemiology and Biostatistics, School of Public Health, Mongolian National University of Medical Sciences, Ulaanbaatar, Mongolia; 4 Department of Restorative Dentistry, School of Dentistry, Mongolian National University of Medical Sciences, Ulaanbaatar, Mongolia

**Keywords:** Oral cancer, Squamous cell carcinoma, Recurrence, Risk factors, Survival rate

## Abstract

**Introduction:**

In Mongolia, there has been limited research on the posttreatment survival rate, recurrence, and occurrence of oral cancer. The goal of this study is to investigate the risk factors that contribute to the recurrence of oral cancer to increase survival rates, facilitate early detection, and improve treatment accuracy.

**Method:**

A retrospective cohort method was used, with medical records from 173 patients diagnosed with squamous cell carcinoma of the mouth at the National Cancer Center of Mongolia’s Department of Head and Neck Surgery, Radio, and Chemotherapy between 2012 and 2017. The Mongolian National University of Medical Sciences’ Research Ethics Committee approved the project.

**Results:**

The findings revealed that 109 cases (63.0%) were men and 64 (37.0%) were females, with a large proportion of patients (28.3%) falling between the ages of 61 and 70. Men had a 3.8 times higher risk of cancer recurrence than women (OR = 3.79, CI = 1.24–11.57). Furthermore, lymph node metastases and treatment were linked to oral cancer recurrence.

**Conclusion:**

This study offers light on the factors that influence the recurrence of oral cancer, giving useful insights for improving patient outcomes through early detection and proper treatment.

## Introduction

Oral cancer is a major global health concern with varying incidence rates across different locations. South and Central Asian nations with the greatest incidence rates of oral cancer, such as India, Sri Lanka, and Pakistan, have a ratio of 100,000 cases per 13.3 persons Rates in Central and Eastern Europe are likewise relatively high, ranging from 100,000:6.2 to 100,000:9.2. Similarly, Australia and New Zealand have a high incidence rate, with a 100,000:8.5 ratio. Excessive alcohol and tobacco consumption, the habit of chewing tobacco and nuts, HPV (16, 18, 33) infection, and exposure to ultraviolet radiation from sunlight are all factors that contribute to these elevated rates [1–5].

The lips, buccal mucosa, alveolar ridge, gingiva, retromolar trigone, floor of the mouth, palate, salivary glands, and the anterior two-thirds of the tongue are all affected by oral cancer [6, 7]. It results from the uncontrolled proliferation of abnormal cells and, if not detected and treated early, can have severe consequences. Recurrence, which is defined as the reappearance of cancer following initial treatment, is a significant obstacle in the management of oral cancer [8].

Identifying the factors associated with oral cancer recurrence is essential for optimizing treatment outcomes, increasing patient survival, and devising targeted interventions. These variables include tumor characteristics, patient demographics, treatment modalities, lifestyle variables, and genetic susceptibility [1–3, 9–16]. The recurrence of oral cancer is a serious obstacle to patient survival rates, influencing prognosis and treatment outcomes [15, 17]. A cohort study, which analyzed data from 83 patients diagnosed with oral squamous cell carcinoma at Patel Hospital in Karachi, discovered a high rate of disease recurrence among T4 patients treated with surgery and adjuvant therapy, with factors such as positive margins and cervical node metastasis influencing outcomes [18]. Another multiple regression analysis revealed that only the existence of a positive surgical margin or a tumor depth larger than 5 mm was substantially linked with recurrence [19]. The researchers discovered that tumors with characteristics such as recurrence, high grade, and high nodal ratio were associated with an increased risk of disease recurrence in this retrospective study of 191 patients with stage III-IVb squamous cell carcinoma of the oral cavity (SCC-OC) who received postoperative radiotherapy (RT) or chemoradiotherapy (CRT) between 1995 and 2013 [20]. However, data on posttreatment survival rates, recurrence, and the occurrence of oral cancer in particular regions, like Mongolia, remains limited [21]. Understanding the risk factors that contribute to the recurrence of oral cancer is critical for improving patient outcomes through early detection, appropriate therapy, and effective management of recurring cases.

As a result, the current study intends to look at the risk factors for oral cancer recurrence in Mongolia. The key goals are to discover specific risk factors and their impact on oral cancer recurrence, as well as to fill research gaps in posttreatment survival rates and recurrence patterns. By attaining these goals, this study hopes to advance our understanding of oral cancer recurrence and provide useful insights for improving patient care and treatment choices in Mongolia.

## Materials and methods

### Study design

The study used a retrospective cohort design to explore the factors influenced on oral cancer recurrence in patients with squamous cell carcinoma of the mouth. The study population included 173 patients diagnosed with squamous cell carcinoma of the mouth and treated at the National Cancer Center of Mongolia’s Department of Head and Neck Surgery, Radio, and Chemotherapy between 2012 and 2017.

#### Study inclusion criteria

To identify study participants, the following inclusion criteria were used.

- Biopsies revealed that the patients had oral cancer.
- Patients who were not treated for the original tumor site in another institution.
- Patients who have had no previous history of malignant tumors in any other region of their body.

### Data collection

The data for this study came from medical reports and records of qualified patients. The following variables were gathered and examined:

- Gender: Either male or female is recorded.
- Age: The patient’s age at the time of diagnosis.
- The location of the primary tumor site in the oral cavity, as described by the International Classification of Diseases for Oncology (ICD-10) categories.
- Histopathologic Grade: The tumor’s grade as determined by histological examination.
- The TNM stage of oral cancer, as defined by the American Joint Committee on Cancer Guide (7th edition).
- Consumption of Alcohol and cigarettes: The presence or absence of alcohol and cigarette use.
- Combination Therapy: Whether the patient underwent multiple therapy techniques.
- Cervical Lymph Node Metastasis: The presence or absence of cervical lymph node metastasis.

### Data analysis

Statistical analysis was carried out STATA statistical data analysis software. To summarize the characteristics of the study population, descriptive statistics were used. Most of the study risk factors were collected as categorical variables are represented as percentages and frequencies. To assess independent risk variables for oral cancer recurrence, univariable and multivariable logistic regressions were performed, and odds ratios were calculated. All statistical tests were two-sided, and P-values less than 0.05 were considered significant. For all odds ratios, we provided 95% confidence intervals.

### Ethical considerations

The study methodology, together with the amended consent process, underwent a thorough evaluation and received approval from the Research Ethics Committee at by the Mongolian National University of Medical Sciences’ Research Ethics Committee. The committee approved the study (Approval No. 2021/3–07) to preserve patients’ privacy, confidentiality, and informed consent.

As the study exclusively relied on pre-existing medical records and did not involve any form of intervention or direct interaction with participants, the ethics committee deemed it unnecessary to get individual participant consent.

## Study results

Table 1 includes a total of 173 instances of oral cancer. There were 109 (63.0%) males and 64 (37.0%) females among these cases. 28.3% of the patients were between the ages of 61 and 70. During the study period, 26 individuals (15%) had a recurrence of oral cancer. Females had a considerably lower recurrence rate (6.3%) than males (20.2%) (P = 0.013). Patients over the age of 61 had a greater recurrence rate, although the difference was not statistically significant. Patients who did not have lymph node metastases had a considerably lower rate of recurrence (94.4%) than those who did (P = 0.009). The cancer recurrence was also observed to be significantly affected by the existence of distant metastases and clinical stages (P = 0.023 and P = 0.047, respectively). Recurrence occurred in 15 (23.4%) of the individuals identified in stage IV. There was a significant difference in treatment options between the recurrence and non-recurrence groups (P = 0.001), with 103 patients (93.6%) who underwent surgery not experiencing cancer recurrence. Cancer site, pathological grading, and tumor size, on the other hand, had no effect on cancer recurrence.

Gender, lymph node metastases, and treatment were found to be substantially linked with oral cancer recurrence in univariable analysis. Men had a 3.8 times higher risk of recurrence than women (OR = 3.79, CI = 1.24–11.57). Patients with numerous lymph node metastases exhibited a 4.5-fold increased risk of recurrence (OR = 4.48, CI = 1.01–19.99). When compared to surgery alone, the combination of surgery plus radiotherapy and surgery plus radiotherapy and chemotherapy increased the risk of recurrence (OR = 8.17, CI = 2.15–31.05 and OR = 14.71, CI = 4.43–48.85, respectively).

**Table 1:** Univariable analyses of risk factors for oral cancer recurrence. (N = 173)

		Cancer recurrence				P value*	cOR^&^	95% CI
		No		Yes				
		Count	Percent	Count	Percent			
**Age**								
	≤ 60 years	73	86.9%	11	13.1%	0.489	1	
	> 60 years	74	83.1%	15	16.9%		1.34	(0.58–3.12)
**Gender**								
	Female	60	93.8%	4	6.3%	0.013	1	
	Male	87	79.8%	22	20.2%		3.79	(1.24–11.57)
**Residence**								
	Urban	65	85.5%	11	14.5%	0.856	1	
	Rural	82	84.5%	15	15.5%		1.08	(0.46–2.51)
**Education**								
	Advanced	35	79.5%	9	20.5%	0.149	1	
	None	5	100.0%	0	0.0%		*	*
	Basic	20	76.9%	6	23.1%		1.17	(0.36–3.76)
	Intermediate	74	91.4%	7	8.6%		0.38	(0.13–1.07)
	Short-cycle tertiary	13	76.5%	4	23.5%		1.19	(0.31–4.56)
**Tobacco consumption**								
	No	82	84.5%	15	15.5%	0.856	1	
	Yes	65	85.5%	11	14.5%		0.92	(0.39–2.15)
**Alcohol consumption**								
	No	113	86.3%	18	13.7%	0.402	1	
	Yes	34	81.0%	8	19.0%		1.48	(0.59–3.69)
**Tobacco and alcohol use**								
	No	115	85.2%	20	14.8%	0.882	1	
	Yes	32	84.2%	6	15.8%		1.08	(0.39–2.91)
**Chipped tooth**								
	No	134	84.8%	24	15.2%	0.848	1	
	Yes	13	86.7%	2	13.3%		0.86	(0.18–4.05)
**Denture sores**								
	No	128	85.3%	22	14.7%	0.733	1	
	Yes	19	82.6%	4	17.4%		1.22	(0.38–3.94)
**Family history**								
	No	135	86.5%	21	13.5%	0.081	1	
	Yes	12	70.6%	5	29.4%		2.68	(0.85–8.37)
**Precancerous conditions**								
	No	129	84.9%	23	15.1%	0.779	1	
	Leukoplakia	8	80.0%	2	20.0%		1.4	(0.28–7.03)
	Others	10	90.9%	1	9.1%		0.56	(0.07–4.59)
**Cancer location**								
	Lip	22	95.7%	1	4.3%	0.056	1	
	Hard palate	9	100.0%	0	0.0%		*	*
	Tongue	62	78.5%	17	21.5%		6.03	(0.75–48.02)
	Cheek lining	9	90.0%	1	10.0%		2.44	(0.14–43.47)
	Gums	15	100.0%	0	0.0%		*	*
	Floor of the mouth	12	75.0%	4	25.0%		7.33	(0.73–73.24)
	Soft palate	15	93.8%	1	6.3%		1.46	(0.08–25.31)
	Retromolar space	3	60.0%	2	40.0%		14.66	(0.99-215.31)
**Tumor size**								
	T1	19	90.5%	2	9.5%	0.309	1	
	T2	50	90.9%	5	9.1%		0.95	(0.17–5.32)
	T3	45	80.4%	11	19.6%		2.32	(0.47–11.49)
	T4	33	80.5%	8	19.5%		2.31	(0.44–11.98)
**Lymph node**								
	N0	34	94.4%	2	5.6%	0.009	1	
	N > 1	91	79.1%	24	20.9%		4.48	(1.01–19.99)
	NX	22	100.0%	0	0.0%		*	*
**Metastasis**								
	M0	121	88.3%	16	11.7%	0.023	1	
	M1	2	50.0%	2	50.0%		7.56	(0.99–57.47)
	MX	24	75.0%	8	25.0%		2.52	(0.97–6.55)
**Stage**								
	2	24	96.0%	1	4.0%	0.047	1	
	1	11	100.0%	0	0.0%		*	*
	3	63	86.3%	10	13.7%		3.81	(0.46–31.38)
	4	49	76.6%	15	23.4%		7.34	(0.92–58.94)
**Pathological grading**								
	Well	111	84.1%	21	15.9%	0.251	1	
	Moderate	5	100.0%	0	0.0%		*	*
	Poor	28	90.3%	3	9.7%		0.57	(0.16–2.03)
	Undifferentiated	3	60.0%	2	40.0%		3.52	(0.55–22.39)
**Treatment**								
	Surgery	103	93.6%	7	6.4%	< 0.001	1	
	CT	4	80.0%	1	20.0%		3.68	(0.36–37.48)
	RT	2	100.0%	0	0.0%		*	*
	Surgery + CT	12	80.0%	3	20.0%		3.68	(0.84–16.14)
	Surgery + RT	9	64.3%	5	35.7%		8.17	(2.15–31.05)
	CT + RT	7	87.5%	1	12.5%		2.1	(0.22–19.56)
	Surgery + CT + RT	9	50.0%	9	50.0%		14.71	(4.43–48.85)
**Rehabilitation**								
	Yes	67	89.3%	8	10.7%	0.160	1	
	No	80	81.6%	18	18.4%		1.88	(0.77–4.61)

^*^ Chi-square test

^&^ Logistic regression analysis

After correcting for important variables identified in the crude analysis, individuals who received surgery with radiotherapy had a greater risk of recurrence than those who received surgery alone (aOR = 11.03, CI = 1.95–62.51). Similarly, individuals who got surgery as well as chemotherapy and radiotherapy had a greater risk of recurrence than those who received only surgery (aOR = 22.53, CI = 3.30-153.7). There were no significant relationships between the probability of recurrence with age, gender, the number of lymph node metastases, clinical stage, or cancer location (Table 2).

**Table 2:** Multivariable analyses of risk factors for oral cancer recurrence. (N = 173)

Characteristics		Odds ratio*	95% CI
Age			
	≤ 60 years	1	
	> 60 years	1.22	(0.08–1.56)
**Gender**			
	Female	1	
	Male	2.79	(0.64–12.22)
**Cancer location**			
	Lip	1	
	Hard palate	*	*
	Tongue	9.96	(0.86-115.86)
	Cheek lining	3.78	(0.12-122.79)
	Gums	*	*
	Floor of the mouth	7.71	(0.45–131.1)
	Soft palate	0.43	(0.01–12.91)
	Retromolar space	14.66	(0.58-369.15)
**Lymph node**			
	N0	1	
	N > 1	5.09	(0.37–69.12)
	NX	*	*
**Stage**			
	1	*	*
	2	1	
	3	0.72	(0.01–36.84)
	4	1.11	(0.02–60.25)
**Treatment**			
	Surgery	1	
	CT	0.79	(0.07–9.04)
	RT	*	*
	Surgery + CT	1.94	(0.35–10.84)
	Surgery + RT	11.03	(1.95–62.51)
	CT + RT	0.76	(0.07–7.75)
	Surgery + CT + RT	22.53	(3.30-153.7)

* Multivariable logistic regression analysis; CI Confidence Interval,

## Discussion

The tongue was the most common site of oral tumors in our study, followed by the lips, soft palate, mouth floor, alveolar ridge, and buccal mucosa. Hard palate cancer and retromolar trigone were the two most common kinds of cancer observed. These findings are consistent with prior research, such as a retrospective cohort study, which found a significant prevalence of buccal mucosa and tongue cancer. In comparison to their findings, our study found a higher prevalence of tongue cancer and a lower prevalence of buccal mucosa cancer [22]. In our study, we discovered comparable trends in the occurrence of oral squamous cell carcinoma locations as the Jinnah Hospital Center in Karachi, Pakistan. There were, however, minor changes in the distribution of tumor locations. Buccal mucosa and gum cancers were more common in our analysis, accounting for a higher proportion of cases, while tongue cancer was less common [23].

The study included [24] that cancer occurred in the tongue (42.2%, 27 cases), gum (17.2%, 11 cases), buccal mucosa (15.6%, 10 cases), floor of the mouth (10.9%, 7 cases), retromolar trigone (7.8%, 5 cases), and hard palate (6.2%, 4 cases), which is consistent with our findings.

According to a [25] 2014 study of 114 patients diagnosed with oral cancer at Imam Khomeini Hospital in Tehran, Iran, tongue cancer (55.2%, 63 cases) and floor of mouth cancer(20.1%, 23 cases) compromised most oral cancers, which is higher than our study result (45.7%, 79; 9.2%, 16 cases). Lips and buccal mucosa were affected in 14.9% (17) and 9.6% (11) of the cases, respectively. The rate was comparable to our study [14]. The study included 67 patients with no history of alcohol or tobacco use among 278 patients diagnosed with oral cancer and hospitalized at Zurich Hospital’s maxillofacial surgery department between 1999 and 2008. The alveolar ridge was the most common site of oral cancer, with 22 cases in the mandibular alveolar ridge and 18 cases in the maxillary alveolar ridge [26]. To contextualize our findings, we compared them to those of Dijk.B.A. and M.T. Brands, who evaluated the survival rates of oral cancer patients in the Netherlands between 2006 and 2010. They determined the survival trajectory based on the location of the cancer and reported a 5-year survival rate for tongue cancer of 65%, which is greater than the rate observed in our study. Similarly, they discovered survival rates of 38% for gingival cancer, 53% for cancer of the alveolar ridge, and 57% for cancer of the floor of the mouth, which corresponds to our findings.

In contrast to their findings, our study found a reduced survival rate for palate cancer (67%). In addition, other oral cancer subsites in our study had a survival rate of 67%, which was greater than our findings for the lips (65.2%), buccal mucosa (60%), soft palate (56.3%), and retromolar trigone (60%) [27].

Notably, there was a considerable gender disparity in the clinical stages of oral cancer. Female patients (34.3%) were diagnosed with cancer in the first and second stages, whereas male patients (87.2%) were diagnosed with cancer in the third and fourth stages. This gender versus clinical stage comparison was shown to be statistically significant (P = 0.003). This was consistent with earlier research [28–33]. The link between gender and cancer stage distribution indicates possible gender variations in risk factors, tumor biology, and healthcare-seeking behavior. However, more research is needed to determine the underlying mechanisms underlying this connection.

Furthermore, the distribution of cancer stages differed greatly amongst tumor locations. Patients with late-stage cancer of the buccal mucosa (70%), alveolar ridge (86.6%), floor of mouth (93.8%), and retromolar trigone (100%) outnumbered those with early-stage cancer of the hard palate (77%). There was a statistically significant difference in cancer stage distribution dependent on tumor location (P < 0.001). In our study, cancer recurrence was observed in 15% (26) of the cases. 6.3% of female patients and 20.3% of male patients experienced a recurrence of oral cancer, with a statistically significant difference between gender groups (P = 0.013). The recurrence rate was significantly higher among patients without lymph node metastasis (P = 0.009). We calculated the recurrence rate based on the TNM stage of the cancer and found that 23.4% of patients diagnosed at stage IV had cancer recurrence, which was statistically substantially higher than patients diagnosed at lower TNM stages. Univariable analysis revealed a statistically significant relationship between cancer recurrence and sex, lymph node metastasis, cancer stages, and treatment modifications. Between January 2002 and December 2006, Bo Wang, Shu Zhang, and others analyzed the survival of 275 patients diagnosed with oral cancer and undergoing surgery at the Cancer Institute and Hospital of Tianjin Medical University [15]. The oral cancer recurrence rate was 32.7% (90 cases), which was higher than our study’s outcome of 15% (26 cases). 57% of cancer recurrences (53 cases) occurred in advanced cancer stages (T3 and T4) (P = 0.000). In our study, however, there was no association between cancer stage and cancer recurrence (P = 0.309). In our study, 48.5% (50 cases) of patients with lymph node metastasis experienced cancer recurrence, compared to 23.3% (40 cases) of patients without lymph node metastasis (P = 0.000). Moreover, cancer location was not a risk factor for cancer recurrence (P = 0.966), corroborating our findings (P = 0.056) [10]. Recurrence occurred in 18.2% (40) of total participants, with 19.5% (17) in the late stage, which is similar to our study results (15% (26), 18.2% (25)). Our study found that 4% (1) of cancer recurrences were in the early stages, but this study found that 17.3% (23) of cancer recurrences were in the early stages. Cancer returned in 19 patients with lymph node involvement after surgical treatment. Patients with positive nodes had 2.88 times greater chances (OR = 2.88, p = 0.01) than patients with negative nodes. Patients with lymph node involvement had 4.5 times the odds of recurrence of oral cancer (OR = 4.48 P = 0.009) than those without lymph node involvement in our research. those in the late stage (T3 and T4) had a 2.33 times greater chance of cancer recurrence than those in the early stage. This discovery is consistent with our previous findings that advanced stage cancer had a 2.33 times higher recurrence risk than early stage cancer [16].

### Study limitations

Our research on oral cancer has shed light on the prevalence of tumor sites, clinical stages, and treatment strategies. Nonetheless, it is essential to recognize the limitations inherent to our study, which may influence the interpretation of the results.

- First, our investigation was conducted at a single center, which may limit the results’ applicability to a larger population. The patient population and healthcare practices at our institution may differ from those of other regions, which may influence the observed distribution of tumor sites, clinical stages, and treatment modalities.
- Second, our study’s retrospective design relied on the analysis of existing medical records and data, which may contain insufficient or absent information. This could potentially incorporate bias or inaccuracy into the data collection procedure.
- Lastly, our study did not account for certain unmeasured confounding factors, such as lifestyle behaviors, socioeconomic status, and comorbidities. These variables may introduce bias and affect the observed relationships between tumor sites, clinical stages, and treatment approaches.

Despite these limitations, our study provides valuable information regarding oral cancer patterns and treatment methods. It lays the groundwork for future research in this field. To validate and expand upon our findings, additional research with larger, more diverse samples and prospective designs is required.

Further investigation into mechanistic explanations for the observed relationships between identified variables and oral cancer recurrence is required. Tumor aggressiveness, genetic changes, immunological responses, and other underlying biological processes are all possible explanations. Investigating these mechanisms through molecular investigations, genetic profiling, and immunological profiling could help us better understand the disease and potentially find novel treatment targets.

This study’s findings have various clinical implications. Identifying risk factors for oral cancer recurrence can help guide treatment options and surveillance techniques. To lower the risk of recurrence, patients with lymph node metastases and those in advanced stages may require more intense follow-up and adjuvant therapy. Gender-specific approaches to oral cancer prevention and care should also be investigated to address noted discrepancies between male and female patients.

Future research should concentrate on improving risk classification models for oral cancer recurrence by adding more clinicopathological factors, molecular markers, and genetic profiling. Prospective research into targeted treatments, immunotherapies, and innovative treatment methods could improve outcomes for patients with high-risk characteristics and minimize the incidence of recurrence.

Finally, our research sheds light on the factors that contribute to the recurrence of oral cancer. Despite the limitations, the findings add to existing information and underline the importance of early detection, customized treatment options, and additional research to enhance patient outcomes in the management of oral cancer in Mongolia.

## Data Availability

The datasets generated and/or analyzed during the current study are available in the [Oral Cancer Mongolia Data Open] repository, https://docs.google.com/spreadsheets/d/1OHvszqSrS-Jgxa7omjTHe7JDIeOeUKiy/edit?usp=sharingouid=115610622034469069068rtpof=truesd=true.
